# Liquid Phase Graphene Exfoliation with a Vibration-Based Acoustofluidic Effector

**DOI:** 10.3390/mi14091718

**Published:** 2023-08-31

**Authors:** Yu Liu, Zhaorui Wen, Ziyu Huang, Yuxin Wang, Zhiren Chen, Shen Lai, Shi Chen, Yinning Zhou

**Affiliations:** Joint Key Laboratory of the Ministry of Education, Institute of Applied Physics and Materials Engineering, University of Macau, Avenida da Universidade, Taipa, Macau 999078, China; yc27812@umac.mo (Y.L.); yb97821@um.edu.mo (Z.W.); yc37827@umac.mo (Z.H.); wangyuxinchn@163.com (Y.W.); yc37817@connect.um.edu.mo (Z.C.); laishen@um.edu.mo (S.L.); shichen@um.edu.mo (S.C.)

**Keywords:** liquid phase exfoliation, graphene, capillary sonication, acoustic streaming, cavitation

## Abstract

Liquid phase exfoliation (LPE) has emerged as a promising method for the industrial-scale production of graphene. However, one of its critical steps, namely sonication, has faced challenges due to high power consumption and low efficiency, leading to limited applicability in industrial settings. This study introduces a novel, cost-effective microfluidic sonication device designed to significantly reduce power consumption while efficiently assisting the LPE process for graphene production. By coupling a capillary with a buzzer and applying an appropriate electric signal, simulation and particle tracing experiments reveal the generation of robust shear forces resulting from acoustic streaming and cavitation when the capillary end is immersed in the liquid. For the first time, the capillary-based sonication device was effectively utilized for graphene exfoliation in a DMF (N,N-Dimethylformamide) + NaOH liquid phase system. The SEM (Scanning Electron Microscope) and Raman characterization results corroborate the successful exfoliation of 100 nm with thicknesses below 10 nm graphene sheets from graphite flakes using this pioneering device. The values of $I_{2D}/I_{G}$ increase after processing, which suggests the exfoliation of graphite flakes into thinner graphene sheets. The vibration-based acoustofluidic effector represents a versatile and scalable miniature device, capable of being employed individually for small-batch production, thereby optimizing the utilization of raw 2D materials, particularly in experimental scenarios. Alternatively, it holds the potential for large-scale manufacturing through extensive parallelization, offering distinct advantages in terms of cost-efficiency and minimal power consumption.

## 1. Introduction

Graphene, the first two-dimensional material with a honeycomb lattice of carbon atoms discovered, exists naturally as a stack of multilayer graphene with interlayer forces governed by van der Waals interactions. Since it was mechanically exfoliated from graphite in 2004 [1], graphene has garnered significant attention due to its exceptional physical and electronic properties [2]. Consequently, extensive efforts have been devoted to exploring its applications in optics [3], electronics [4], energy systems [5], biosensors [6], and catalysis [7]. The vast array of applications has resulted in substantial demand for graphene, prompting the exploration of various preparation methods, such as mechanical exfoliation (‘Scotch tape’ method) [8], epitaxial growth [9], liquid phase exfoliation [10], chemical vapor deposition (CVD) [11], and oxidation-reduction techniques [12]. The attainment of industrial-scale graphene production at a low cost continues to be of utmost significance.

Liquid phase exfoliation (LPE) has proven to be a suitable method for achieving the mass production of graphene [13,14,15,16]. In 2008, Coleman et al. [17] successfully dispersed graphite in N-methyl pyrrolidone (NMP) and prepared graphene through sonication. Subsequently, numerous liquid phase systems were introduced to enhance LPE efficiency, including organic solvents (such as NMP, DMF), aqueous solutions, and surfactants [18,19,20]. These liquid phase systems share a common principle: the efficiency of exfoliation is heightened when the ratios of surface tension components of the solvent closely match those of graphene. This equilibrium occurs because the energy required for graphene exfoliation is balanced by the solvent’s interaction with graphene, particularly for solvents whose surface energies align with that of graphene [21,22]. Kai et al. [18] systematically evaluated a wide range of solvents and suggest that the solvent exfoliation efficiency and disperse ability are two separate keys for the yield of graphene produced by LPE. In this study, we have adopted the commonly used liquid phase system comprising DMF and NaOH (as a surfactant) for enhancing exfoliation efficiency and disperse ability. Apart from the appropriate liquid phase system, sonication stands as another crucial factor influencing LPE efficiency. Commonly employed equipment includes sonication baths [23] and sonication probes [24,25,26,27], generating shear forces and cavitation for the exfoliation process. However, considerable energy consumption, inhomogeneous acoustic fields, and low efficiency associated with these methods restrict their applicability in industrial-scale LPE production. As an example, for improving the production rate of graphene, Yoshihiko et al. [27] used a high-power probe sonicator (600 W) and thus obtained a 0.1 g/h high production rate of graphene dispersions in NMP. Furthermore, excessive sonication energy input can lead to the sonolysis of solvent molecules and elevate liquid temperatures, necessitating cooling systems to maintain optimal system conditions. These issues not only decrease production efficiency, but also escalate production costs. Therefore, achieving high shear rates and efficient cavitation while minimizing power consumption holds great appeal for LPE industrial production.

Lab-on-chip and microfluidization techniques present a promising opportunity to meet the demanding requirements of graphene exfoliation [28,29,30]. For instance, Wang et al. [31] utilized a lab-scale pressure-homogenized microfluidizer for the graphene exfoliation process. They introduced a high-pressure graphene suspension into a Z-shaped microchannel, where the resulting high shear force led to the production of graphene sheets. In another study, Qiu et al. [32] reported a low-pressure hydrodynamic cavitating ‘lab on a chip’ for graphene exfoliation. This device was micromachined on a silicon wafer, integrating a microchannel with a micro-step, creating a gap of height of 132 μm within the microchannel. As the suspension passed through the microchannel, high shear force and cavitation were generated, facilitating the production of graphene. Despite the application of microfluidic devices in the liquid exfoliation of graphene, their high cost and complex fabrication processes have limited their widespread use.

In addition to hydrodynamic microfluidics, acoustofluidics devices have also been employed for graphene exfoliation. Ahmed et al. [29] utilized interdigital transducers (IDTs) to induce high-frequency surface acoustic waves, enabling microfluidic nebulization and graphene exfoliation. In acoustofluidics devices, the sharp edge serves as a commonly employed and typical structure within the chip. It facilitates the generation of strong acoustic streaming and cavitation induced by bulk acoustic waves in its vicinity. Numerous studies have demonstrated this phenomenon and its application in micromixers and chemical synthesis [33,34,35,36,37,38]. However, capillaries can serve as a viable substitute for the sharp edge structure inside acoustofluidics chips. Durrer et al. [39] have already demonstrated the potential of capillary-based acoustofluidics end effectors for fluid pumping and trapping embryos. These findings indicate that sharp edge-based capillaries may prove advantageous in the liquid phase exfoliation of graphene.

In this study, we introduce a novel and highly efficient sonication device designed for exfoliating natural graphite into graphene sheets within a liquid phase system. The device primarily comprises a microneedle (capillary) and a buzzer, securely bonded together using UV light curing glue. Its low cost and ease of assembly render it a practical and promising choice. Through acoustic streaming simulations and fluorescence particle tracing experiments, we have substantiated that the vibrated capillary generates robust acoustic streaming and cavitation effects in the liquid. In the DMF + NaOH liquid system, the capillary-based sonication device with low energy consumption replaces conventional high energy consumption sonication devices (probes and baths) during the sonication process. The characterization of the resulting graphene suspension is performed using Raman and SEM tests, revealing a certain level of effectiveness in the liquid phase exfoliation of graphene by the new device. The miniaturization of the device allows for small-scale production, and its potential for volume production through massive parallelization offers promising avenues for future applications.

## 2. Experimental Methods

### 2.1. Materials

The graphite powder used in the experiments was purchased from Aladdin (G123645, 99.95%, metal basis, ≥325 mesh, Shanghai, China) and used without further treatment. DMF (N, N-Dimethylformamide) were provided by Macklin (N807505, AR, 99.5%, Shanghai, China). NaOH (Sodium hydroxide) came from SCR (Sinopharm Chemical Reagent Co., Ltd., 10019762, Shanghai, China). DMF was chosen because its surface tension value is 37.5 $mjm^{-2}$, which is very close to the graphene for overcoming the van der Waals force to break the bond [40]. NaOH was chosen as the surfactant to assist the exfoliation process because it brings stability to the final graphene dispersion [41].

### 2.2. Sonication Device

**Figure 1:**
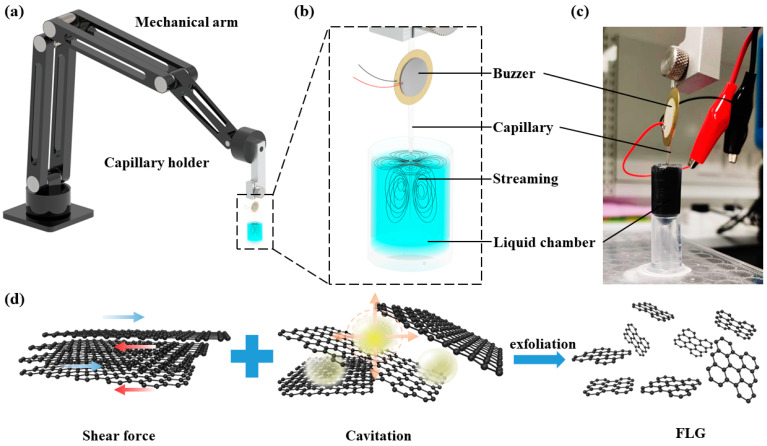
(**a**) The schematic of capillary-based liquid phase exfoliation system/sonication system. (**b**) Schematic of capillary vibration induced streaming. (**c**) The actual setup picture of capillary vibration induced streaming. (**d**) Main principle of sonication-assisted exfoliation process; under the combination effect of shear force and cavitation, graphite flakes were exfoliated to produce few layers graphene.

The novel capillary-based liquid phase exfoliation system (Figure 1a) consists of a mechanical arm, a customized capillary holder, a buzzer and a glass capillary. The buzzer has a 10 mm diameter piezoelectric ceramic coupled with a 20 mm diameter brass plate, and the thickness is 0.2 mm. The resonance frequency of the buzzer is 3 kHz for sound production, but the practical frequency range used in this experiment was 17.5–18.5 kHz for sonication. The specific frequency chosen was based on acoustic streaming and the cavitation effect in the liquid system. The glass capillary had a 1 mm outer diameter at its base and was pre-pulled, creating a narrow tip of only 3 μm diameter at its end (MPI-0100, WEIKE Co., Ltd., Wuhan, China). The buzzer was bonded using a UV light curing glue (ergo 8500, Kisling AG, Zurich, Switzerland) at 2 cm above the capillary tip and was excited by an arbitrary function generator (AFG 31000, TEKTRONIX, Inc., Beaverton, OR, USA) using the sine wave (amplitudes range: 0~10 volt peak to peak ($V_{pp}$), frequencies range: 0~1 MHz). The output sine waves were amplified by a high voltage amplifier (WMA-300, Falco Systems BV, Katwijk aan Zee, The Netherlands) for a stronger sonication effect. The mobile mechanical arm makes the capillary moveable and tightly fixed in a desired position. The capillary tip was immersed in a cylindrical liquid chamber for the sonication process and was placed at the center of the chamber to ensure minimum boundary effects during sonication and streaming experiments.

### 2.3. Exfoliation Mechanism

As illustrated in Figure 1b, the buzzer generates acoustic waves based on the input signal from a function generator that regulates frequency and amplitude, thereby controlling the oscillation of the capillary. The actual setup of the capillary end effector is shown in Figure 1c. When the immersed capillary undergoes oscillations within the liquid, its tip (sharp edge) generates intricate vortices in the surrounding medium, a phenomenon commonly known as acoustic streaming. Acoustic streaming is a nonlinear acoustic effect which converts acoustic energy to fluid flow [36]. When the capillary tip (sharp edge) is excited, the steep velocity gradient induces inner streaming inside the boundary layer, and then outer streaming vortices will generate in the nearby bulk fluids. High-aspect-ratio (sharp edge) structures exhibit stronger streaming compared to low-aspect-ratio structures at the same acoustic intensity level. Consequently, a capillary with a micrometer tip was selected as the sonication end effector due to its ability to produce more robust acoustic streaming effects. Steady 3D streaming with distinct profiles developed close to the capillary end. Generally, four symmetric vortices appear parallel to the horizontal plane. Flow to/flow out symmetric streaming appeared in the vertical to horizontal plane (Figure 1b). Cavitation is another phenomenon that is induced by sharp edge oscillations and produced in the sonication process. When the capillary tip oscillation drastically, negative acoustic pressure reached a certain level, which vaporized the liquid for forming cavities in the liquid. At high acoustic power, voids or bubbles will violently oscillate to rapidly collapse and produce shock waves. Bubble collapse can provide strong mechanical energy.

During the exfoliation process, graphene can be peeled from the pristine graphite layer by layer. As the complex acoustic streaming generated from the capillary tip, shear force produced and overcame the van der Waals force between layers, in which FLG (Few Layers Graphene) could be peeled down due to their lateral self-lubricating ability. On the other hand, the cavitation-induced bubble can result in shock waves, which will produce normal and shear forces in graphite, and some microbubbles may come into the space between layers and expand the interlayer spacing [42,43]. All these effects from sonication will lead to a more effective exfoliation process, as shown in Figure 1d.

### 2.4. Acoustic Streaming Simulation Mechanism and Methods

The acoustic streaming simulation was conducted for better understanding and for the optimization of the sonication process. The detailed calculation process based on the perturbation method is provided below, which provides a theoretical foundation for the subsequent simulation.

With the external acoustic actuation, liquid perturbations can be described by three variables: temperature T, pressure p  and velocity v. Taking the first and second order (subscripts 1 and 2) into account, the variables can be expressed as:

(1) $$T=T_{0}+T_{1}+T_{2}$$

(2) $$p=p_{0}+p_{1}+p_{2},$$

(3) $$v=v_{1}+v_{2},$$

where $T_{0}$ and $p_{0}$ are the constant temperature and pressure before excitation, and v=0 on all walls.

The acoustic source is molded as the boundary condition using the first order velocity $v_{1}$, expressed as:

(4) $$n\cdot v_{1}=v_{a}e^{-i\omega t},$$

where n is the normal vector, $v_{a}$ is the normal velocity magnitude at the actuated boundary and ω is the angular frequency characterizing the harmonic time dependence.

The first-order acoustic field can be obtained by employing the thermos-viscous acoustic module in the frequency domain. The first order equation can be expressed as:

(5) $$i\omega T+\gamma D\nabla^{2}T=\frac{\gamma -1}{\alpha }\nabla \cdot v_{1},$$

(6) $$i\omega \rho_{f}v_{1}+\mu \nabla^{2}v_{1}+\mu \left[\beta +i\frac{1}{\gamma k\mu \omega }\right]\nabla \left(\nabla \cdot v_{1}\right)=\frac{\alpha }{\gamma k}\nabla T,$$

where γ is the specific heat capacity ratio, *D* is the thermal diffusivity, $\rho_{f}$ is the fluid density, μ is the fluid dynamic viscosity, k is the fluid compressibility and β is the viscosity ratio.

The second-order acoustic can be obtained by employing the laminar flow module based on the calculated $v_{1}$ and $\rho_{1}$ in the first order acoustic fields:

(7) $$\rho_{f}\nabla \cdot \langle v_{2}\rangle =-\nabla \cdot \langle \rho_{1}v_{1}\rangle ,$$

(8) $$\mu \nabla^{2}\langle v_{2}\rangle +\beta \mu \nabla \left(\nabla \cdot \langle v_{2}\rangle \right)-\langle \nabla p_{2}\rangle =\langle \rho_{1}\partial_{t}v_{1}\rangle +\rho_{f}\langle \left(v_{1}\cdot \nabla \right)v_{1}\rangle .$$

The vibration-induced acoustic streaming flow fields around the capillary tip were simulated using a finite element analysis software COMSOL Multiphysics 6.0. Based on necessary assumptions for simplification, we considered the capillary tip to be a rigid oscillating cone with base radius a = 10 μm. The surrounding fluid domain was large enough (radius = 30 a) to avoid the boundary effect.

The first order acoustic fields are assumed to be time-harmonic and calculated with the thermos-viscous acoustic module. The capillary tip boundary is prescribed as a Dirichlet boundary condition with a known displacement profile. Once the first order flow fields have been solved, the source terms in second-order equations are identified and a laminar flow module with a steady state can be studied. We refer the reader to a prior study [44] for further detailed discussion on governing equations and numerical simulation.

### 2.5. Particle Tracing Experiment Mechanism and Method

The related particle tracing experiments were studied using inverted fluorescent microscopy (IX73, Olympus Corporation, Tokyo, Japan). The device was characterized by immersing the capillary tip into a cylindrical liquid chamber containing 5 μm blue fluorescence tracer particles (polystyrene granule, Rigor science, Wuxi, China) mixed with DIW: F127 (0.6 wt%) in a volume ratio of 1:100. The tip was positioned at the chamber’s center to avoid boundary effects as much as possible during the characterization of the particle trace. The continual image was captured using a high-resolution camera (DP74, Olympus Corporation, Tokyo, Japan). 

The working principle of the particle tracing behavior is based on the acoustic radiation force, arising from the scattering of acoustic waves on the microparticle, and the drag force induced by acoustic streaming. For the situation of the manipulation of microparticles smaller than the acoustic wavelength (a << λ), the ARF on particles can be expressed as:

(9) F=−∇U,

where *U* presents Gor’kov force potential and is expressed using the acoustic pressure and velocity as:

(10) $$U=(4\pi a^{3}/3)\left[f_{1}\langle p^{2}\rangle /2\rho_{0}c_{0}^{2}-{3f}_{2}\rho_{0}\langle {\left|v\right|}^{2}\rangle /4\right],$$

where $4\pi a^{3}/3$ is the volume of the particle and the scattering coefficients are given by:

(11) $$f_{1}=1-\rho_{0}c_{0}^{2}/\rho_{p}c_{p}^{2},\;{\;f}_{2}=2\left(\rho_{p}-\rho_{0}\right)/\left(2\rho_{p}+\rho_{0}\right),$$

where $\rho_{p}$ and $c_{p}$ are the density and the longitudinal wave velocity of the particle. The scattering coefficients $f_{1}$ and $f_{2}$ represent the monopole and dipole coefficients, respectively.

The suspended particles in the fluid will also experience a drag force from the acoustic streaming, which is given by:

(12) $$F_{D}=6\pi \mu au,$$

where μ is the dynamic viscosity and u is the velocity of surrounding flows relative to the particle. Under influence from ARF and drag force, microparticles will follow the stream and show the streaming pattern through fluorescence.

### 2.6. Exfoliation Method and Characterization Process

Graphite powder was added into DMF with NaOH as the surfactant to assist the exfoliation. The initial concentration of graphite was 30 mg/mL, and the concentration of NaOH was 10 mg/mL (not all NaOH was dissolved). The exfoliation process was performed via sonication over 9 h. The capillary-based sonication device with a 3 μm tip was immersed in the 2 mL solution. The excitation signal from the function generator was 18.8 kHz and 1 $V_{pp}$, and then the signal went through a 50X voltage amplifier to the buzzer. After sonication, the dispersions were collected for further centrifugation. The centrifugation parameters were 4000 rpm and 1 h. After centrifugation, the large and thick flakes settled down and the exfoliated sheets remained in the solution. The top 80% of the supernatant was collected for further characterization. 

The surface morphology of the natural graphite and the FLG sheets were characterized using a Scanning Electron Microscope (Sigma FESEM, Carl Zeiss AG, Oberkochen, German) at a voltage of 2 kV [21]. The samples were prepared by drop casting supernatants on a clean Si wafer (cleaned by ethanol and dried) and dried on hotplate at 150 °C, then put into the drying oven for 24 h to remove the solvent. The sample was coated with gold for clearer detection.

For the Raman test, the graphene dispersions were drop-casted onto a 285 nm $SiO_{2}/Si$ wafer and dried on a hotplate at 150 °C. Raman spectroscopy was performed using the Micro Raman system (LABHRev-UV, HORIBA Scientific, Kyoto, Japan). An objective lens of ×100, a laser wavelength of 532 nm and a laser intensity of 100% were used to acquire the Raman spectra.

## 3. Results and Discussions

### 3.1. Streaming

In this section, we will describe the acoustic streaming developed near the capillary tip. At first, a 3D acoustofluidics simulation model was built, and the streaming pattern and velocity under different vibration mode was simulated. To display the streaming under the acoustic field, blue fluorescence microparticles were diluted and suspended in 0.6% wt F127 buffered solution and placed into a reservoir (~200 μL). The capillary tip was close to the reservoir bottom for clearer characterization using an inverted fluorescence microscope.

**Figure 2:**
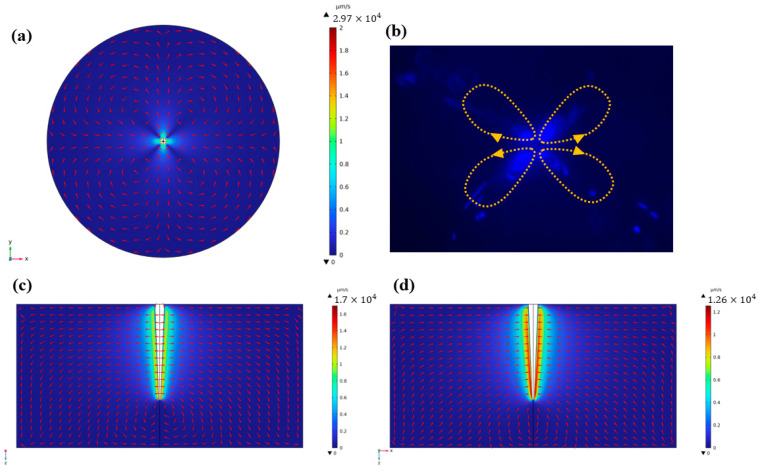
(**a**) Acoustic streaming simulation. The capillary vibrates in the y direction and generates four symmetry vortexes in the x–y plane. (**b**) Particle tracing image with four symmetry vortexes; (**c**,**d**) acoustic streaming simulation with two symmetry vortexes in the y–z and x–z planes.

The acoustic streaming phenomenon was initially simulated under a frequency of 18.1 kHz, with the vibration direction along the y-axis. As depicted in Figure 2a, the red cone illustrates the streaming pattern, revealing four symmetrical vortices formed near the capillary in the x–y plane. The streaming emanates from the x-axis and subsequently returns to the y-axis, with the capillary serving as the origin point. The distribution of velocity also shows that the maximum flow velocity appears close to the capillary boundary. With the superposition of two adjacent vortexes, the flow velocity is higher along the x and y axis. In the y–z plane and the x–z plane, the direction of the streaming is determined by the vibration direction of the capillary, with the streaming either entering or emanating from the capillary. Furthermore, the velocity is consistently at its peak in close proximity to the capillary, as indicated in Figure 2c,d. As mentioned above, the simulation results reveal a significant streaming effect resulting from capillary vibration. The velocity distribution also shows a great decline along the direction away from capillary, which indicated that shear force can generate from acoustic streaming and assist the peeling of graphene from graphite. These simulation outcomes were further validated through particle tracing experiments. When the capillary was activated with an excitation frequency of 2.9 kHz and amplitude of 300 mVpp, its oscillations attracted the tracer particles, causing them to follow the flow field trajectories. This resulted in the manifestation of four symmetrical vortices around the capillary on the horizontal plane, as depicted in Figure 2b.

**Figure 3:**
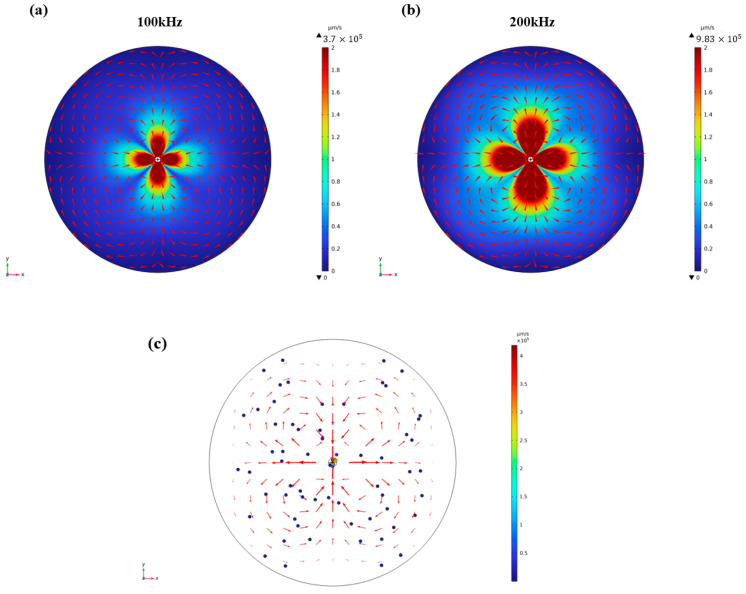
Acoustic streaming velocity and pattern in (**a**) 100 kHz and (**b**) 200 kHz. The simulation results show that four symmetry vortexes are generated in the x–y plane, and both the maximum velocity value and high velocity distribution area increase with the frequency. (**c**) Particle tracing trajectories’ simulation results.

**Figure 4:**
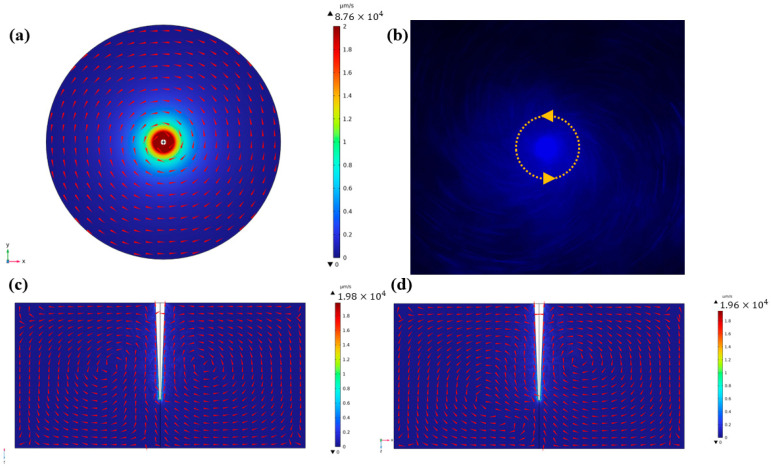
Acoustic streaming simulation. (**a**) capillary vibrations in the x and y direction with 90° phase difference. Circular vortex generated around capillary in x–y plane; (**b**) particle tracing image with circular vortex; and (**c**,**d**) acoustic streaming simulation with two symmetry vortexes in the y–z and x–z planes.

The strength of the flow field produced by the capillary is determined by the intensity of the acoustic wave, which was demonstrated by adjusting the frequency applied to the capillary in the simulation. In the simulation model, the vibration velocity of the capillary is governed by the frequency, as the amplitude of the capillary boundary remains fixed for simplicity. This implies that the frequency exhibits a positive correlation with the intensity of the acoustic wave. The streaming velocity distribution simulation results for 18.1 kHz (Figure 2a), 100 kHz (Figure 3a) and 200 kHz (Figure 3b) were compared on the horizonal plane. It is worth noting that the scale of the velocity value was the same for comparative purposes. The results reveal that both the maximum velocity value and the area with high velocity distribution increased with the frequency, indicating the intensity of the acoustic wave. In the experimental setup, the intensity of the acoustic wave was determined by the amplitude of the signal voltage. However, due to limitations in the properties of the buzzer and the strength of the capillary, the maximum value of the voltage was set at 2 Vpp. This voltage level was found to be sufficient for generating strong acoustic streaming in the small reservoir. The particle tracing trajectories’ simulation results are also shown in Figure 3c. The red arrow presents acoustic streaming velocity. The arrow length is proportional to the streaming velocity. It is obvious that streaming produces a higher velocity in the junction of vortexes. The color of particle presents the speed of it. The results show that a particle has the highest speed near the capillary. And some particles were trapped by the capillary, although most of the particles moved following streaming. This can explain how strong blue fluorescence occurs near the capillary in experiments (Figure 2b and Figure 4b).

**Figure 5:**
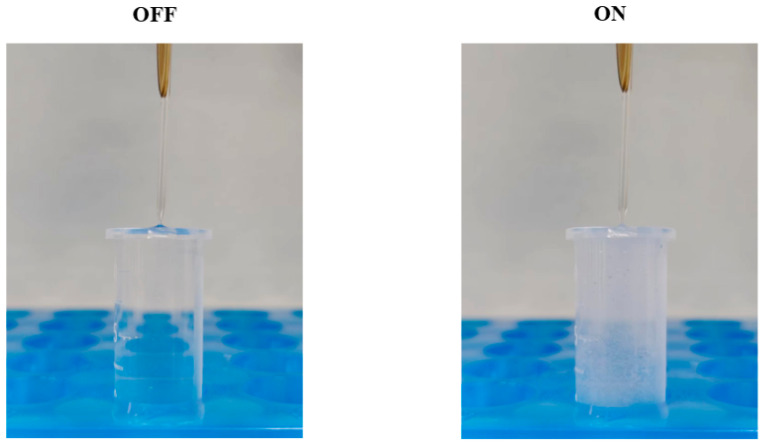
Cavitation phenomenon when the capillary is immersed in liquid and actuated. When the buzzer becomes excited, the capillary tip starts vibrating vigorously, creating microbubbles that then flow along with the stream, resulting in a turbid appearance of the liquid.

In addition to the unidirectional vibration, simulations were conducted with x- and y-direction vibrations with a 90° phase difference, as depicted in Figure 4. In the x–y plane, the streaming pattern exhibited a counterclockwise motion around the capillary, with the velocity attaining its maximum value in proximity to the capillary boundary (Figure 4a). Furthermore, two symmetrical vortices associated with the capillary were observed in all planes perpendicular to the x–y plane. The direction of streaming converged toward the capillary, while the maximum velocity was generated in the close vicinity of the capillary (Figure 4c,d). This phenomenon was further confirmed by the particle tracing experiments. In Figure 4b, the microparticles were trapped near the capillary and rotated counterclockwise around the capillary under 12.1 kHz and 300 mVpp. And it is obvious that more particles were trapped near the capillary due to round high-speed flow generation. The variations in the streaming pattern with frequency can be elucidated by changes in the vibration model. Drawing insights from Jan’s study [39], it is evident that the capillary tip exhibits distinct vibration modes at different frequencies. Specifically, under the frequency of 2.9 kHz, the capillary tip underwent translational motion, while at 12.1 kHz it exhibited elliptical motion. This kind of acoustic streaming should be avoided for overconcentrated flow velocity distribution, as its presence has the potential to adversely influence the efficiency of graphene exfoliation. The significant influence of cavitation in experiments cannot be disregarded, as it is considered the main principle underlying the graphene exfoliation process. The selection of excitation signal parameters during the sonication process was based on acoustic streaming and cavitation phenomena. As illustrated in Figure 5, microbubbles are generated and follow the streaming when the buzzer is excited at 18.1 kHz and 2 Vpp, and they vanish when the excitation signal is turned off.

As mentioned before, we analyzed simulation and experimental results about acoustic streaming and cavitation generating from a vibration-based capillary end effector. By choosing appropriate excitation signals, the shear force from strong acoustic streaming and cavitation can be applied in a small container for graphene liquid phase exfoliation. Detailed experiments have already been mentioned in Section 2.6. The below part will characterize the graphene product after the experiment with SEM and the Raman test.

### 3.2. SEM Observation

**Figure 6:**
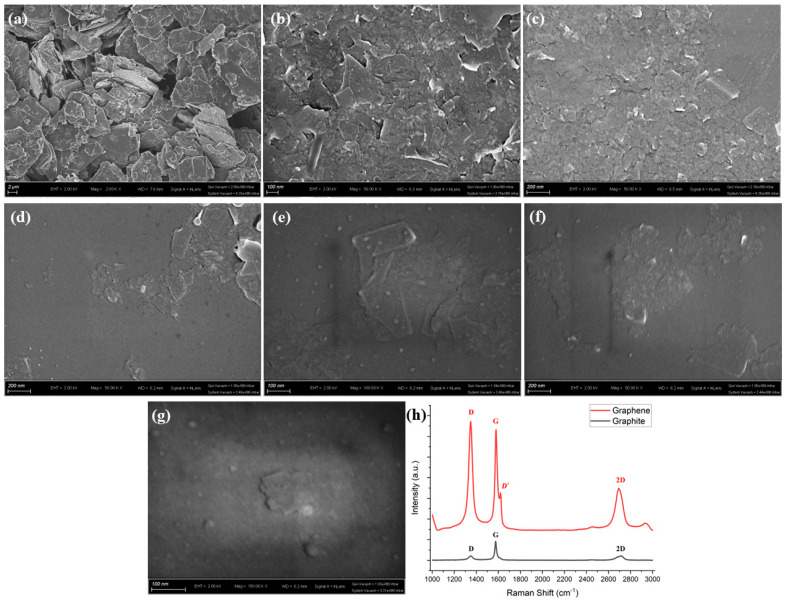
SEM characterization results of (**a**) graphite powder, (**b**,**c**) graphene sheets overlapped, (**d**–**g**) single graphene sheets and (**h**) Raman spectroscopy comparison of graphite and graphene.

Figure 6a shows that the pristine graphite powder consists of polydisperse flakes with a lateral size below 10 μm and a thickness below 1 μm. In comparison, the SEM observation of the graphene dispersion samples has demonstrated that the liquid phase exfoliation with the sonication-assisted process had a significant impact on the graphite powders. It is obvious from Figure 6b,c that nano graphene sheets overlapped and generated a thin graphene film under the drop-casting method. The clear border in SEM images may result from the accumulation of charges on the electrical insulation surfactant [32]. Furthermore, as shown in Figure 6d–g, it becomes evident that the average size of the graphene sheet is much smaller than that of the graphite flakes, with lateral sizes below 100 nm and thicknesses below 10 nm. The SEM observations corroborate the notion that the sonication process effectively fragments and exfoliates the graphite flakes into nano graphene sheets.

### 3.3. Raman Spectroscopy

Raman spectroscopy was deployed for scrutinizing the quality of graphene, including defects, layer count and exfoliation evidence [24]. The main three characteristic peaks, D peak (~1350 $cm^{-1}$), G peak (~1580 $cm^{-1}$) and 2D peak (~2600 $cm^{-1}$), were recorded. The defects in graphene are usually characterized by the ratio of intensities for D and G bands ($I_{D}/I_{G}$) [16]. Principally, the variations in intensity, peak position, and width of the 2D band reveal information related to the graphene layer count [45,46].

Figure 6h displayed the spectra recorded from a pristine graphite flake (black) and from a processed graphene sheet (red), respectively. In comparison, the G band’s full width at half maximum (FWHM) of graphene was smaller than graphite. The ratio of amplitude of the 2D and G peak ($I_{2D}/I_{G}$) increased after processing, indicating the exfoliation of graphite flakes into thinner graphene sheets. However, the ratio of amplitude of the D and G peak ($I_{D}/I_{G}$) increased and exceeded 1 after processing, suggesting the generation of edge defects and basal defects during the exfoliation process. The defect peak $D^{\prime }$ also appeared (~1620 $cm^{-1}$), which meant serious defects generated in graphene sheets. Moreover, although the ideal $I_{2D}/I_{G}>1$ ratio was not observed in the final samples, this does not imply that the graphite was not exfoliated. In reality, Figure 6b,c provide a clear explanation for this observation. The Raman test employed a laser spot diameter of 1 μm, resulting in the characterization of numerous nanosheets of graphene, which contributes to the increase in defect peaks. It is inevitable that the sonication process induces the fragmenting of flakes, leading to a reduction in their size. The re-aggregation of graphene into a film is the key reason behind the absence of prominently high $I_{2D}/I_{G}$ ratios.

### 3.4. Discussion

Along with these experiments, we proved that graphene sheets can be extraordinarily exfoliated in solution by using a vibration-based capillary effector. Through acoustic streaming simulation and particle tracing experiments, we found that strong mechanical shearing, acoustic cavitation, and microbubbles can be generated by a vibration-based capillary effector in a small container. To give the pros and cons of the new microfluidic device clearly, we compared commonly used assist devices in the LPE process in Table 1.

As in Table 1, the pros of the capillary effector are: (i) low power consumption; (ii) low cost and easy fabrication. Cons still exist. After processing, graphene sheets are too small because of the strong acoustic streaming and cavitation not just peeling off the graphene, but also tearing the bulk graphite into pieces. However, based on LPE studies using a sonication probe, the qualification of graphene can be raised by optimizing parameters like the excitation signal, input power, sonication time and capillary depth to the liquid surface. Indeed, a solitary capillary-based sonication apparatus exhibits capability in generating robust acoustic streaming, thereby facilitating Liquid Phase Exfoliation (LPE) within a confined volume. Nonetheless, the economic viability and energy efficiency intrinsic to such miniaturized devices offer compelling prospects for their integration into industrial paradigms. Firstly, our projections posited the realization of large-scale graphene manufacturing through the deployment of automated parallelization mechanisms. Secondly, the concurrent production of diverse, layered materials essential for experimentation could remarkably amplify production efficiency, particularly in scenarios demanding parallel detection. Thirdly, the inherent advantages of miniaturization equip the device with seamless adaptability into automated production lines, underscoring its industrial utility. Furthermore, this device exhibits applicability in contexts demanding robust acoustic streaming and cavitation, such as in the domains of mixing and chemical synthesis.

## 4. Conclusions

This study introduces a novel method for graphene liquid phase exfoliation, employing a capillary-based sonication approach for the first time. The main conclusions are listed as follows:

(1) By coupling a low-cost buzzer and microcapillary using UV curing glue and applying a low-consumption electric signal, a promising simple yet effective sonication device was developed.
(2) Acoustic streaming simulations and particle tracing experiments were conducted by immersing the capillary tip in the liquid, revealing significant acoustic streaming generated near the capillary tip, which increased with the intensity of the acoustic waves.
(3) Additionally, at 18.1 kHz, cavitation phenomena were observed in the liquid chamber, characterized by the generation of microbubbles that followed the streaming. These phenomena induced shear force and cavitation, and are considered to be the key mechanisms underlying the sonication-assisted graphene exfoliation process.
(4) Sonication-assisted LPE was conducted for 9 h using DMF + NaOH, and the resulting sheets were subjected to analysis using various characterization techniques. SEM and Raman results revealed the production of 100 nm graphene sheets with a thin thickness.

The capillary-based sonication device demonstrated low power consumption and affordability, making it an attractive option for industrial-scale graphene production through the implementation of automatic parallelization devices in the LPE system.

## Figures and Tables

**Table 1:** Comparison of commonly used devices and capillary effector for assisting in the LPE process.

Device	Exfoliation Principle	Power Consumption	Graphene Qualification	Cost/Fabrication Process
Sonication bath [17,23]	Shear force and cavitation	High	Good	High
Sonication probe [24,25,26,27]	Shear force and cavitation	High	Good	High
Microfluidizer [31]	Shear force	Low	Good	High
Microfluidic chip [29,32]	Shear force	Low	Good	High/complex
Capillary effector (This study)	Shear force and cavitation	Low	100 nm sheets	Low/easy

## Data Availability

Data will be available upon request.

## References

[1] Novoselov K.S., Geim A.K., Morozov S.V., Jiang D., Zhang Y., Dubonos S.V., Grigorieva I.V., Firsov A.A. (2004). Electric field effect in atomically thin carbon films. Science.

[2] Novoselov K.S., Fal′Ko V.I., Colombo L., Gellert P.R., Schwab M.G., Kim K. (2012). A roadmap for graphene. Nature.

[3] Vakil A., Engheta N. (2011). Transformation Optics Using Graphene. Science.

[4] Ratnikov P.V., Silin A.P. (2018). Two-dimensional graphene electronics: Current status and prospects. Phys. Uspekhi.

[5] Li Y., Yang J., Song J. (2016). Nano energy system model and nanoscale effect of graphene battery in renewable energy electric vehicle. Renew. Sustain. Energy Rev..

[6] Garg M., Pamme N. (2023). Microfluidic (bio)-sensors based on 2-D layered materials. TrAC Trends Anal. Chem..

[7] Wang B., Sun Q., Liu S., Li Y. (2013). Synergetic catalysis of CuO and graphene additives on TiO_2_ for photocatalytic water splitting. Int. J. Hydrogen Energy.

[8] Yi M., Shen Z. (2015). A review on mechanical exfoliation for the scalable production of graphene. J. Mater. Chem. A.

[9] Yang W., Chen G., Shi Z., Liu C.-C., Zhang L., Xie G., Cheng M., Wang D., Yang R., Shi D. (2013). Epitaxial growth of single-domain graphene on hexagonal boron nitride. Nat. Mater..

[10] Qian M., Zhou Y.S., Gao Y., Feng T., Sun Z., Jiang L., Lu Y.F. (2012). Production of few-layer graphene through liquid-phase pulsed laser exfoliation of highly ordered pyrolytic graphite. Appl. Surf. Sci..

[11] Asif M., Tan Y., Pan L., Li J., Rashad M., Usman M. (2015). Thickness Controlled Water Vapors Assisted Growth of Multilayer Graphene by Ambient Pressure Chemical Vapor Deposition. J. Phys. Chem. C.

[12] Wan Y., Chen L., Tang W., Li J. (2020). Effect of graphene on tribological properties of Ni based composite coatings prepared by oxidation reduction method. J. Mater. Res. Technol..

[13] Xu Y., Cao H., Xue Y., Li B., Cai W. (2018). Liquid-Phase Exfoliation of Graphene: An Overview on Exfoliation Media, Techniques, and Challenges. Nanomaterials.

[14] Bari R., Parviz D., Khabaz F., Klaassen C.D., Metzler S.D., Hansen M.J., Khare R., Green M.J. (2015). Liquid phase exfoliation and crumpling of inorganic nanosheets. Phys. Chem. Chem. Phys..

[15] Liu Y., Li R. (2019). Study on ultrasound-assisted liquid-phase exfoliation for preparing graphene-like molybdenum disulfide nanosheets. Ultrason. Sonochem..

[16] Alaferdov A., Gholamipour-Shirazi A., Canesqui M., Danilov Y., Moshkalev S. (2013). Size-controlled synthesis of graphite nanoflakes and multi-layer graphene by liquid phase exfoliation of natural graphite. Carbon.

[17] Hernandez Y., Nicolosi V., Lotya M., Blighe F.M., Sun Z., De S., McGovern I.T., Holland B., Byrne M., Gun’Ko Y.K. (2008). High-yield production of graphene by liquid-phase exfoliation of graphite. Nat. Nanotechnol..

[18] Ng K.L., Maciejewska B.M., Qin L., Johnston C., Barrio J., Titirici M.-M., Tzanakis I., Eskin D.G., Porfyrakis K., Mi J. (2022). Direct Evidence of the Exfoliation Efficiency and Graphene Dispersibility of Green Solvents toward Sustainable Graphene Production. ACS Sustain. Chem. Eng..

[19] Ciesielski A., Samori P. (2014). Graphene via sonication assisted liquid-phase exfoliation. Chem. Soc. Rev..

[20] Smith R.J., King P.J., Lotya M., Wirtz C., Khan U., De S., O’Neill A., Duesberg G.S., Grunlan J.C., Moriarty G. (2011). Large-Scale Exfoliation of Inorganic Layered Compounds in Aqueous Surfactant Solutions. Adv. Mater..

[21] Coleman J.N., Lotya M., O’Neill A., Bergin S.D., King P.J., Khan U., Young K., Gaucher A., De S., Smith R.J. (2011). Two-Dimensional Nanosheets Produced by Liquid Exfoliation of Layered Materials. Science.

[22] Qian W., Hao R., Hou Y., Tian Y., Shen C., Gao H., Liang X. (2009). Solvothermal-assisted exfoliation process to produce graphene with high yield and high quality. Nano Res..

[23] Liu W., Tanna V.A., Yavitt B.M., Dimitrakopoulos C., Winter H.H. (2015). Fast Production of High-Quality Graphene via Sequential Liquid Exfoliation. ACS Appl. Mater. Interfaces.

[24] Morton J.A., Kaur A., Khavari M., Tyurnina A.V., Priyadarshi A., Eskin D.G., Mi J., Porfyrakis K., Prentice P., Tzanakis I. (2023). An eco-friendly solution for liquid phase exfoliation of graphite under optimised ultrasonication conditions. Carbon.

[25] Sethurajaperumal A., Varrla E. (2022). High-Quality and Efficient Liquid-Phase Exfoliation of Few-Layered Graphene by Natural Surfactant. ACS Sustain. Chem. Eng..

[26] Shen J., He Y., Wu J., Gao C., Keyshar K., Zhang X., Yang Y., Ye M., Vajtai R., Lou J. (2015). Liquid Phase Exfoliation of Two-Dimensional Materials by Directly Probing and Matching Surface Tension Components. Nano Lett..

[27] Arao Y., Kubouchi M. (2015). High-rate production of few-layer graphene by high-power probe sonication. Carbon.

[28] Jafarpour M., Aghdam A.S., Gevari M.T., Koşar A., Bayazıt M.K., Ghorbani M. (2021). An ecologically friendly process for graphene exfoliation based on the “hydrodynamic cavitation on a chip” concept. RSC Adv..

[29] Ahmed H., Rezk A.R., Carey B.J., Wang Y., Mohiuddin M., Berean K.J., Russo S.P., Kalantar-Zadeh K., Yeo L.Y. (2018). Ultrafast Acoustofluidic Exfoliation of Stratified Crystals. Adv. Mater..

[30] Choi C.-H., Kwak Y., Malhotra R., Chang C.-H. (2020). Microfluidics for Two-Dimensional Nanosheets: A Mini Review. Processes.

[31] Wang Y.-Z., Chen T., Gao X.-F., Liu H.-H., Zhang X.-X. (2017). Liquid phase exfoliation of graphite into few-layer graphene by sonication and microfluidization. Mater. Express.

[32] Qiu X., Bouchiat V., Colombet D., Ayela F. (2019). Liquid-phase exfoliation of graphite into graphene nanosheets in a hydrocavitating ‘lab-on-a-chip’. RSC Adv..

[33] Shafaghi A.H., Talabazar F.R., Zuvin M., Gevari M.T., Villanueva L.G., Ghorbani M., Koşar A. (2021). On cavitation inception and cavitating flow patterns in a multi-orifice microfluidic device with a functional surface. Phys. Fluids.

[34] Hao N., Liu P., Bachman H., Pei Z., Zhang P., Rufo J., Wang Z., Zhao S., Huang T.J. (2020). Acoustofluidics-Assisted Engineering of Multifunctional Three-Dimensional Zinc Oxide Nanoarrays. ACS Nano.

[35] Zhao S., He W., Ma Z., Liu P., Huang P.-H., Bachman H., Wang L., Yang S., Tian Z., Wang Z. (2019). On-chip stool liquefaction via acoustofluidics. Lab Chip.

[36] Liu Y., Yin Q., Luo Y., Huang Z., Cheng Q., Zhang W., Zhou B., Zhou Y., Ma Z. (2023). Manipulation with sound and vibration: A review on the micromanipulation system based on sub-MHz acoustic waves. Ultrason. Sonochemistry.

[37] Zhou Y., Wang H., Ma Z., Yang J.K.W., Ai Y. (2020). Acoustic Vibration-Induced Actuation of Multiple Microrotors in Microfluidics. Adv. Mater. Technol..

[38] Athanassiadis A.G., Ma Z., Moreno-Gomez N., Melde K., Choi E., Goyal R., Fischer P. (2021). Ultrasound-Responsive Systems as Components for Smart Materials. Chem. Rev..

[39] Durrer J., Agrawal P., Ozgul A., Neuhauss S.C.F., Nama N., Ahmed D. (2022). A robot-assisted acoustofluidic end effector. Nat. Commun..

[40] Güler Ö., Tekeli M., Taşkın M., Güler S.H., Yahia I. (2020). The production of graphene by direct liquid phase exfoliation of graphite at moderate sonication power by using low boiling liquid media: The effect of liquid media on yield and optimization. Ceram. Int..

[41] Liu W.W., Wang J.N. (2011). Direct exfoliation of graphene in organic solvents with addition of NaOH. Chem. Commun..

[42] Telkhozhayeva M., Teblum E., Konar R., Girshevitz O., Perelshtein I., Aviv H., Tischler Y.R., Nessim G.D. (2021). Higher Ultrasonic Frequency Liquid Phase Exfoliation Leads to Larger and Monolayer to Few-Layer Flakes of 2D Layered Materials. Langmuir.

[43] Han J.T., Jang J.I., Kim H., Hwang J.Y., Yoo H.K., Woo J.S., Choi S., Kim H.Y., Jeong H.J., Jeong S.Y. (2014). Extremely Efficient Liquid Exfoliation and Dispersion of Layered Materials by Unusual Acoustic Cavitation. Sci. Rep..

[44] Muller P.B., Barnkob R., Jensen M.J.H., Bruus H. (2012). A numerical study of microparticle acoustophoresis driven by acoustic radiation forces and streaming-induced drag forces. Lab Chip.

[45] Malard L.M., Pimenta M.A., Dresselhaus G., Dresselhaus M.S. (2009). Raman spectroscopy in graphene. Phys. Rep..

[46] Shimada T., Sugai T., Fantini C., Souza M., Cançado L., Jorio A., Pimenta M., Saito R., Grüneis A., Dresselhaus G. (2005). Origin of the 2450 cm^−1^ Raman bands in HOPG, single-wall and double-wall carbon nanotubes. Carbon.

